# PPARγ Attenuates Cellular Senescence of Alveolar Macrophages in Asthma- COPD Overlap

**DOI:** 10.21203/rs.3.rs-4009724/v1

**Published:** 2024-03-05

**Authors:** Rongjun Wan, Prakhyath Srikaram, Shaobing Xie, Qiong Chen, Chengping Hu, Mei Wan, Yuanyaun Li, Peisong Gao

**Affiliations:** Johns Hopkins University School of Medicine; Johns Hopkins University School of Medicine; Johns Hopkins University School of Medicine; Central South University; Central South University; Johns Hopkins University School of Medicine; Central South University; Johns Hopkins University School of Medicine

**Keywords:** ACO, Asthma, COPD, Macrophages, Senescence, PPARγ

## Abstract

Asthma-chronic obstructive pulmonary disease (COPD) overlap (ACO) represents a complex condition characterized by shared clinical and pathophysiological features of asthma and COPD in older individuals. However, the pathophysiology of ACO remains unexplored. We aimed to identify the major inflammatory cells in ACO, examine senescence within these cells, and elucidate the genes responsible for regulating senescence. Bioinformatic analyses were performed to investigate major cell types and cellular senescence signatures in a public single-cell RNA sequencing (scRNA-Seq) dataset derived from the lung tissues of patients with ACO. Similar analyses were carried out in an independent cohort study Immune Mechanisms Severe Asthma (IMSA), which included bulk RNA-Seq and CyTOF data from bronchoalveolar lavage fluid (BALF) samples. The analysis of the scRNA-Seq data revealed that monocytes/ macrophages were the predominant cell type in the lung tissues of ACO patients, constituting more than 50% of the cells analyzed. Lung monocytes/macrophages from patients with ACO exhibited a lower prevalence of senescence as defined by lower enrichment scores of SenMayo and expression levels of cellular senescence markers. Intriguingly, analysis of the IMSA dataset showed similar results in patients with severe asthma. They also exhibited a lower prevalence of senescence, particularly in airway CD206 + macrophages, along with increased cytokine expression (e.g., *IL-4, IL-13,* and *IL-22*). Further exploration identified alveolar macrophages as a major subtype of monocytes/macrophages driving cellular senescence in ACO. Differentially expressed genes related to oxidation-reduction, cytokines, and growth factors were implicated in regulating senescence in alveolar macrophages. PPARγ (Peroxisome Proliferator-Activated Receptor Gamma) emerged as one of the predominant regulators modulating the senescent signature of alveolar macrophages in ACO. Collectively, the findings suggest that senescence in macrophages, particularly alveolar macrophages, plays a crucial role in the pathophysiology of ACO. Furthermore, PPARγ may represent a potential therapeutic target for interventions aimed at modulating senescence-associated processes in ACO.

## INTRODUCTON

Asthma and chronic obstructive pulmonary disease (COPD) are two of the most prevalent obstructive lung diseases. Asthma-COPD overlap (ACO) is a term used to describe a complex condition characterized by shared clinical and pathophysiological features of both diseases, typically observed in older individuals with a long history of disease and exposure to environmental factors^1–3^. ACO is estimated to affect approximately 10–30% of individuals with asthma and around 25% of those with COPD^4^. Cumulative environmental exposures, such as exposure to allergens and toxic particles or gases (e.g., smoking and indoor biomass), are believed to play a crucial role in the development and progression of ACO^1,5^. Additionally, asthma and atopy have been identified as potential risk factors for the development of COPD and, consequently, ACO^6^. More importantly, patients with ACO often experience increased rates of exacerbations and clinical symptoms, posing a significant challenge for clinical treatment and management^7,8^. However, managing ACO patients can be intricate due to the overlapping nature of these diseases.

The inflammatory mechanisms in asthma and COPD are distinct. In asthma, inflammation is primarily driven by Th2 cell-mediated responses, leading to eosinophilic inflammation and an increase in cytokines like IL-4, IL-5, and IL-13^9^. On the other hand, COPD is characterized by inflammation with a higher presence of neutrophils and a dominance of Th1 and Th17 cell-mediated responses ^10^. In both conditions, macrophages, the primary immune cells in the lungs, play crucial roles in the immune response, defense against infections, tissue homeostasis, and inflammation resolution^11^. Macrophages in asthma and COPD can contribute to airway inflammation and remodeling by releasing pro-inflammatory cytokines, proteases, and reactive oxygen species^12,13^. Therefore, it is reasonable to believe that macrophages also play a vital role in the inflammatory processes and pathophysiology of ACO.

ACO is more commonly diagnosed in older individuals, and its prevalence tends to increase with age, as supported by various studies^14,15^. Age has been identified as a significant risk factor for ACO in asthma patients^16^. Consequently, the aging process and senescence appear to play substantial roles in the development of lung inflammation in individuals with ACO. Senescence represents a complex cellular state characterized by cellular stress, DNA damage, cell cycle arrest, and the release of senescence-associated secretory phenotype (SASP) factors^17–19^. These SASP factors encompass chemokines, cytokines, growth factors, adhesion molecules, and lipid components that can contribute to multiple age-related disorders with both local and systemic consequences^20–27^. It’s worth noting that cellular senescence has been associated with both asthma^28,29^ and COPD^30–32^. Consequently, senescence may exert a significant influence on the development and management of ACO. However, it is essential to recognize that senescence is a complex process influenced by factors such as cell type, age, and specific diseases^29,33^. Therefore, it is crucial to investigate whether senescence in lung macrophages contributes to the inflammatory processes and pathophysiology unique to ACO.

In our current research, we conducted an analysis using publicly available single-cell RNA sequencing (scRNA-Seq) data obtained from human lung tissues, comparing individuals with and without ACO. Our primary focus was on identifying the major cell types and identified monocytes/macrophages as the predominant cell types in patients with ACO. We delved into gene signatures associated with senescence, specifically within monocytes/macrophages. The findings were further validated through another independent cohort (IMSA) with a specific emphasis on investigating the relationship between cellular senescence features and the severity of asthma. Additionally, we explored differentially expressed genes and pathways within alveolar macrophages of individuals with ACO and identified PPARγ as a key regulatory factor in driving cellular senescence in alveolar macrophages.

## METHODS

### Data Source

The scRNA-Seq data was publicly available and was generated from human lung tissues of a patient with ACO who died with exacerbation and two transplant donors^34^. The Bulk RNA-Seq and mass cytometry (cytometry by time of flight, CyTOF) data were derived from bronchoalveolar lavage fluid (BALF) samples and downloaded from the Gene Expression Omnibus (GEO) database with the accession number GSE136587, and the Flow Repository (FR) database with the identifier FR-FCM-Z395^35^. The data were collected as a part of the Immune Mechanisms Severe Asthma (IMSA) study and involved the analysis of bronchoscopically obtained distal airway and alveolar cells. The study included a total of 39 subjects consisting of 6 healthy controls, 17 mild/moderate asthma patients, and 16 severe asthma patients.

### Single Cell RNA-Seq Processing and Analyzing

#### Quality Control

The raw counts matrixes downloaded were loaded to R workspace to initiate a Seurat workflow. A series of critical preprocessing steps were performed to ensure data quality before the analysis. Specifically, cells with a count of unique molecular identifiers (UMIs) below 500, cells expressing fewer than 200 genes with a log10GenesPerUMI value below 0.75, and cells with mitochondrial gene expression contributing to over 20% of the total gene expression were removed.

#### Dimension Reduction and Cell Annotation

Following the quality control procedures, the processed data underwent integration using distinct strategies: the complete dataset was integrated utilizing Seurat^36^, while the Monocytes/Macrophages subset was integrated using the Harmony approach^37^. The dimensionality reduction and visualization were achieved using the Uniform Manifold Approximation and Projection (UMAP) algorithm. UMAP not only captures inter-cellular distances but also provides a holistic view of the data’s global structure^38^. Cell clusters were identified by Leiden algorithm, an automated algorithm tailored for effectively clustering cells in scRNA-seq data^39^. Cell markers were these with differentially expressed genes (DEGs) identified by the FindAllMarkers function from the Seurat package. Cellular annotations were identified by SingleR or manually based on specific marker expression, which allows us to get the composition and dynamics of cells within the dataset^40^.

#### Differential Expressing Analyses

Differential expressing analyses were conducted using the Libra R package (Version 1.0.0). Within this framework, the “run_de” function was employed, applying Wilcoxon rank sum tests to assess statistical differences between groups. Genes with an adjusted P value < 0.05 and absolute log2-fold change (|log2FC|) > 0.5 were termed as statistical significance. Moreover, the Seurat package’s “FindMarkers” and “FindAllMarkers” functions were also utilized to identify markers that distinguish cell clusters with Wilcoxon rank sum tests.

#### Enrichment Analyses

The enrichment analyses included geneset scoring through the AUCell algorithm, and over-representation analysis (ORA). The AUCell algorithm was employed to gauge cellular senescence enrichment. This algorithm calculated an enrichment score by juxtaposing the input data against the SenMayo senescence geneset, an optimal choice for senescence screening since it consistently aligns with results from biological validation experiments^41^. The ORA analysis function RunEnricher was embedded within the High-Dimensional Weighted Gene Co-Expression Network Analysis (hdWGCNA) R package. It was employed to investigate the biological characteristics of selected co-expression modules.

#### Trajectory Analyses

A partition-based graph abstraction (PAGA) based trajectory analysis was used to investigate the trajectory of monocytes/macrophages within single-cell RNA sequencing (scRNA-seq) data. This procedure was performed using Scanpy version 1.9.1 in Python 3.10.9., which revealed the intricate relationships and transitions among different cell states. This workflow simplified the dataset’s graph representation by clustering similar cells, enabling the identification of connected and disconnected regions. By combining well-defined paths rooted in high-confidence connections and leveraging a random-walk-based distance metric, cells are systematically ordered within each partition according to their proximity to a designated reference cell^42^.

#### High-Dimensional Weighted Gene Co-Expression Network Analysis (hdWGCNA)

To explore the hub genes and the underlying expression network associated with cellular senescence and related biological processes, we harnessed the power of hdWGCNA (Version 0.2.18). This algorithm is a refinement of the traditional WGCNA designed specifically for scRNA-Seq data. It operates by computing a sparsity-reduced expression matrix termed metacell, akin to the concept of pseudobulk to mitigate the influence of undetected genes and facilitate the identification of gene co-expression modules with clinical or biological information^43,44^

#### Transcription Factor Analyses

Transcription factors are crucial for the initial stage of decoding DNA sequencing and regulation of gene expression^45^. To initiate this process, we employed PySCENIC (Version 0.12.1)^46^ to identify cell cluster-specific transcription factors. Moreover, we extended our investigation by extracting a gene list from the cellular senescence-related gene module identified using hdWGCNA. Subsequently, this gene list was subjected to transcription factor enrichment analysis using the Transcriptional Regulatory Relationships Unraveled by Sentence-based Text mining (TRRUST) database on the metascape website (metascape.org)^47,48^.

### Bulk RNA-Seq Processing and Analyzing

#### Data Preprocessing

The expression matrix encompassing raw counts and pertinent phenotype information was procured from the GEO website, facilitated by the GEOQuery R package (Version 2.66.0)^49^. The raw counts with batch details were downloaded for differential expressing analysis. Furthermore, the raw counts were normalized and then transformed into log2-transformed transcripts per million (log2TPM) values. To mitigate the batch-induced discrepancies, the Combat algorithm embedded within the sva R package (Version 3.44.0)^50^.

#### Unsupervised Clustering and Differential Expressing Analyses

Due to heterogeneity of asthma, we did not make direct comparisons for senescence signature between different asthma groups. Instead, we employed an unsupervised clustering approach that focused on the expression profiles of genes associated with cellular senescence^51^. For the clustering process, we utilized the k-means algorithm, which groups data points based on their similarity as measured by the Euclidean distance with the ConsensusClusterPlus R package (Version 1.60.0)^52,53^. The differential expressing analysis was performed to explore the variations in gene expression across these clusters by following the limma-voom approach with batch information adjusted in the model^54^.

### CyTOF Processing and Analyzing

#### Data Preprocessing

An outlier sample displaying an unusually high intensity value of 3 in the live_dead channels was identified and subsequently excluded from the CyTOF analysis. Cells exhibiting extremely low CD45 expression were also filtered out using Flowjo. The resultant fcs data was then transferred to an R workspace for subsequent analysis. To ensure accurate analysis, the data underwent a bead-based normalization process utilizing the “normCytof” function within the CATALYST R package (Version 1.20.1)^55^.

#### Dimension Reduction and Cell Annotation

Similar to scRNA-Seq, dimension reduction for visualization and cell annotation were required for CyTOF core analyses. Especially, the t-Distributed Stochastic Neighbor Embedding (t-SNE) algorithm was employed^56^. For automated cluster identification, the FlowSOM^57^ was used. Cell type annotations were based on expression profiles of major cell type markers similar to that described in relevant publications^35^.

#### Differential Abundance (DA) and Differential States (DS) Analyses Based on CyTOF Data

Differential cell proportion (abundance) and cytokine expression (states) between cellular senescence clusters identified by unsupervised clustering were calculated with diffcyt R package (1.16.0). Both limma based DA (diffcyt-DS-voom), and DS (diffcyt-DA-trend) parameters were chosen for differential expression analyses^54,58^.

#### Statistical Analysis and Data Visualization

All analysis and visualizations were processed with R (Version 4.1.2 for scRNA-Seq and Version 4.2.1 for CyTOF and Bulk RNA-Seq), Python (Version 3.10.9) and GraphPad (Version 9.3). Wilcoxon rank sum tests were employed for continuous variables between two groups; Chi-Square tests were performed for the comparisons of categorical variables. Correlation analyses (Pearson) were based on Single-Cell Variational Inference (scVI-tools, Version 0.20.2) imputed expression data to avoid sparsity influence^59^. Data visualizations were performed with ggplot2 (Version 3.4.1), genekitr (Version 1.0.5), ggVennDiagram (Version 1.2.2) and ComplexHeatmap (Version 2.10.0) R packages.

## RESULTS

### Alveolar Macrophages were the Major Cell types in Patients with ACO

**Figure 1:**
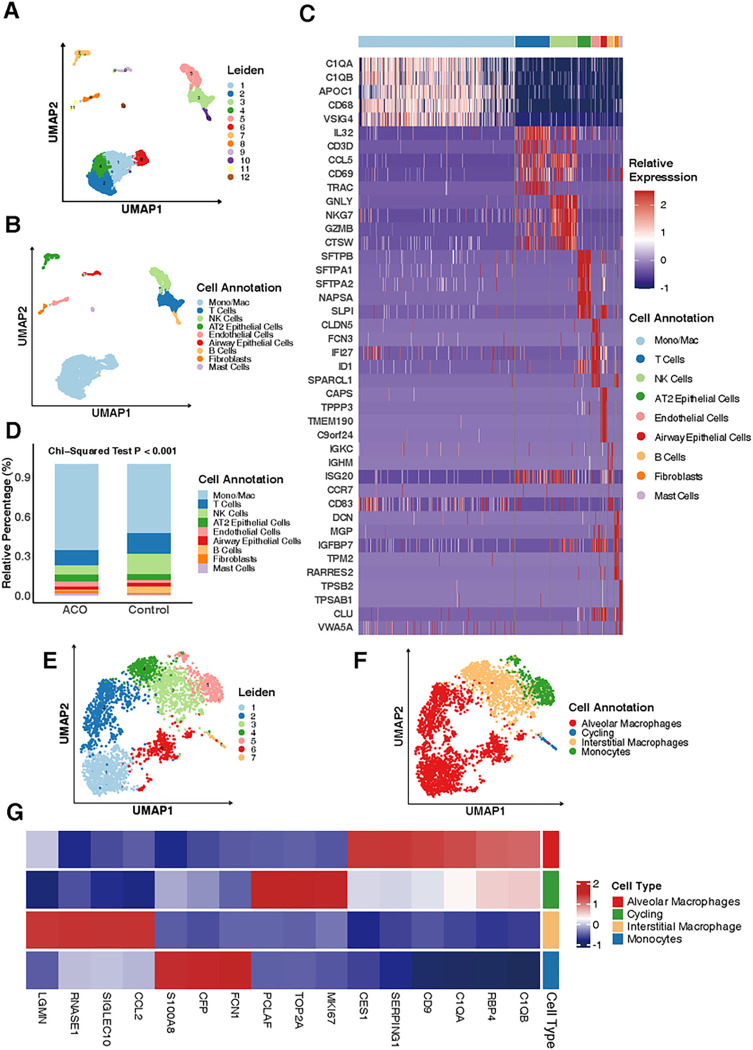
Alveolar macrophages were the major cell types in patients with ACO. **A,** A total of 12 cell clusters were identified within the scRNA-Seq dataset generated from human lung tissues of patients with ACO (n=1) and without ACO (n=2). **B,** Cell clusters in (**A**) were further annotated as different cell types by using the SingleR algorithm. **C,** Heatmap represents the top-ranking differentially expressed genes for each cell type in (**B**). **D**, Relative percentage of each cell type in ACO and controls. **E**, A total of seven clusters were identified within monocytes/macrophages by using the Leiden algorithm. **F**, Cell clusters in (**E**) were further annotated as different sub-types of monocytes/macrophages. **G**, Heatmap represents the top-ranking differentially expressed genes for each cell type in (**F**). ACO: asthma-COPD overlap. Chi-Square tests were performed for the comparisons of category variables.

To better understand the pathogenesis of ACO, we analyzed the existing scRNA-Seq dataset generated from human lung tissues of a patient with ACO and two transplant donors without ACO^34^. By using the Leiden algorithm, an automated algorithm designed to effectively cluster cells in scRNA-seq data^39^, we identified a total of 12 cell clusters within dataset (Fig. 1, A). These cell clusters were further annotated as different cell types by using the SingleR algorithm with manual adjustments. Cell markers were these differentially expressed across different cell types identified by the FindAllMarkers function from the Seurat package^36^. A total of nine different cell types were identified within the dataset, which included monocytes/macrophages, T cells, NK cells, AT2 alveolar type II cells, endothelial cells, airway epithelial cells, B cells, fibroblasts, and mast cells (Fig. 1, B). The top-ranking differentially expressed genes for each cell type were presented in Fig. 1, C and Figure E1). For example, complement-related genes (*C1QA, C1QB*), *CD68*, and *APOC1* were highly expressed in monocytes/macrophages (Figure E1, A), while genes like *SFTPB, SFTPA1,* and *SFTPA2* were uniquely and significantly expressed in AT2 airway epithelial cells ( Figure E1, B). Tryptase genes (*TPSAB1, TPSB2*) were highly expressed in mast cells (Figure E1, C). To identify the distribution of different cell types in the samples from ACO and control group, we performed cell proportion analysis as defined by relative percentages (Fig. 1, D). Of these, monocytes/macrophages were a predominant cell type, constituting more than 50% of the cells in the different groups being analyzed. Furthermore, the proportion of monocytes/macrophages was significantly higher in ACO patient compared to control group (*p* < 0.001). Next, we focused on those monocytes/macrophages specifically and re-clustered them by using the Leiden algorithm. A total of seven clusters were identified (Fig. 1, E). These cell clusters were further annotated as different sub-types of monocytes/macrophages (Fig. 1, F), including alveolar macrophages (AM), cycling cells, interstitial macrophages (IM), and monocytes. The top-ranking differentially expressed genes for each cell type were presented in Fig. 1, G and Figure E2). Similar to monocytes/macrophages, the subtype AMs express the complement-related genes *C1QA, C1QB* ( Figure E2, A). Additionally, several other genes *RBP4, CD9, SERPING1,* and *CES1* are also significantly expressed in AMs. The proliferation markers *PCLAF, TOP2A* and *MKI67* were highly expressed in cycling cells (Figure E2, B). IMs were in an intermediate state in the UMAP plot and expressed high levels of molecules such as *LGMN, RNASE1,* and *CCL2* (Figure E2, C). While *CFP, FCN1,* and *S100A8*,were highly expressed in monocytes (Figure E2, D). Taken together, the results suggest that monocytes/macrophages in ACO are major cells that may drive airway inflammation.

### Decreased cellular Senescence in Monocytes/Macrophages of Patients with ACO

**Figure 2:**
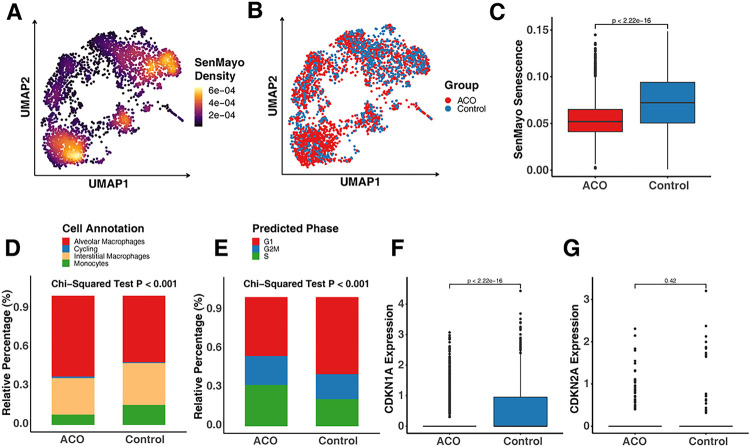
Deceased cellular senescence in monocytes/macrophages of patients with ACO. **A,** Density of AUCell enriched SenMayo senescence score within lung monocytes/macrophages. **B,** Distribution of ACO and control group among monocytes/macrophages. **C,** Enrichment scores of SenMayo senescence in ACO and control group. **D,** Relative percentage of each monocyte/macrophage subtype in ACO and controls. **E,** Relative percentage of each cell cycle phases in ACO and controls. **F-G,** Expression of cellular senescence markers CDKN1A (p21, **F**) and CDKN2A (p16, **G**) in ACO and controls. Chi-Square tests were performed for the comparisons of category variables. Wilcox rank sum tests were used for the comparison of numerical variables. A p < 0.05 was considered as statistical significance.

Cellular senescence has gained considerable attention across various diseases, including respiratory conditions such as asthma^29^. To determine whether senescence contributes to the development of ACO, we specifically assessed the enrichment of genes associated with senescence within lung monocytes/macrophages. Employing the AUCell algorithm, we computed enrichment scores against a senescence-related gene set known as SenMayo^41^. Among all the subtypes, AMs/monocytes showed the higher enrichment sore as assessed by the density of AUCell enriched SenMayo senescence scores (Fig. 2, A). Next, we investigated the distribution of ACO and control group among monocytes/macrophages (Fig. 2, B) and enrichment scores of SenMayo senescence in ACO and control group (Fig. 2, C). As illustrated in Fig. 2, C, the senescent signatures are enriched in the control group as compared to ACO group. Among them, AMs were a predominant cell type, constituting more than 50% of the cells among all these subtypes (Fig. 2, D). The proportion of AMs was significantly higher in ACO patients as compared to control group (*p* < 0.001). Additionally, cell cycle arrested in the G1 phase is one of the important features of cellular senescence^60^. We found that monocytes/macrophages that were in the G1 phase of the cell cycle were more abundant in the control group compared with ACO group (Fig. 2, E). These findings were further supported by the expression of cellular senescence markers, CDKN1A (p21) and CDKN2A (p16)^61^. The expression of CDKN1A was much lower in the ACO group as compared to the control group (Fig. 2, F). In contrast, no significant change was observed for CDKN2A (Fig. 2, G). These findings suggest a lower prevalence of cellular senescence within lung monocytes/macrophages among patients with ACO.

### A lower prevalence of senescence observed in patients with severe asthma

**Figure 3:**
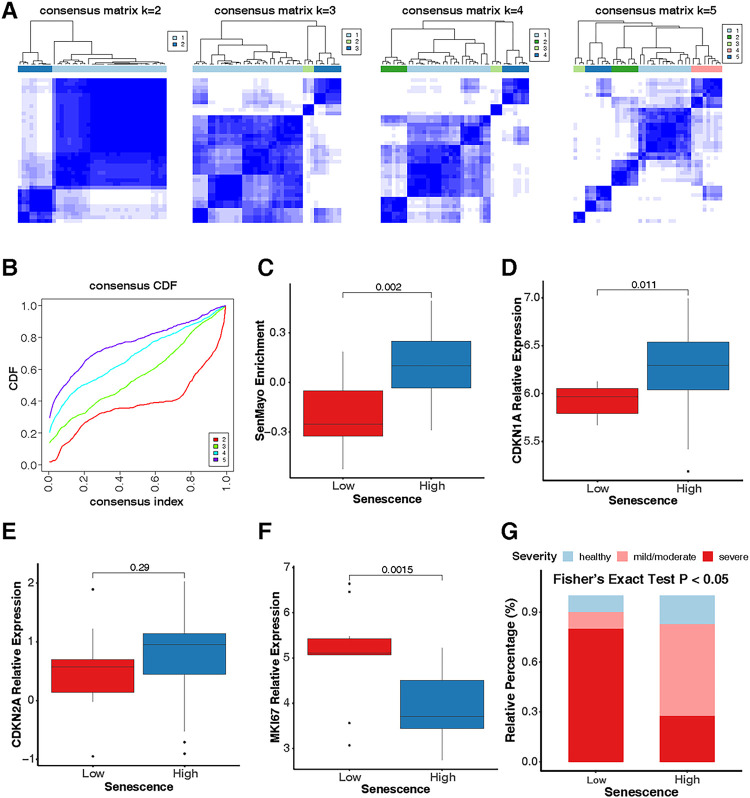
A lower prevalence of senescence in patients with severe asthma in a IMSA dataset. **A,** Identification of stable and meaningful clusters by repeated consensus clustering analysis with different values of k (from 2 to 5) within a IMSA dataset. **B,** Consistent and stable cellular senescence clusters identified by Cumulative Distribution Function (CDF). **C,** Two clusters were assigned senescence low and high group according to the ssGSEA SenMayo Senescence score. **D-F,** Relative expression of senescence markers CDKN1A (p21, **D**), CDKN2A (p16, **E**), and proliferation marker MKI67 (**F**) in senescence clustering low and high group. **G**, Relative percentage of health control (HC, n=5), mild/moderate asthma patients (MMA, n=17), and severe asthma (SA, n=16) participants in senescence clustering low and high group. Fisher’s Exact test was performed for the comparisons of category variables. Wilcox rank sum tests were used for the comparison of numerical variables between 2 groups. A p < 0.05 was considered as statistical significance.

We further validated the results through another independent cohort (IMSA)^35^ and analyzed the relationship between cellular senescence features and the severity of asthma. After stringent quality control, a total of 39 subjects were finally included for analysis: 6 healthy controls (HC), 17 mild/moderate asthma patients (MMA), and 16 severe asthma patients (SA). Definition of these subjects has been previously reported^9^. Consensus clustering analysis with the Consensus Cumulative Distribution Function (CDF) identified cellular senescence clusters for immune cells from IMSA. Consensus clustering analysis involves repeated clustering of the data with different values of k (from 2 to 5) within a dataset to identify stable and meaningful clusters (Fig. 3, A). Based on the consensus CDF (Fig. 3, B), it is evident that cellular senescence clusters 2 provides the most consistent and stable clustering solution for the IMSA dataset. According to the ssGSEA SenMayo Senescence score, the cluster 2 was divided into senescence clustering low and high group (*P* = 0.002, Fig. 3, C). Senescence clustering low group showed lower expression of senescence markers CDKN1A as compared to senescence high group (*P* = 0.011, Fig. 3, D). No difference was noted for CDKN2A between senescence clustering low and high group (*P* = 0.29, Fig. 3, E). In contrast, senescence low group showed higher expression of cell proliferation marker MKI67 (*P* = 0.0015, Fig. 3, F). Intriguingly, among the analyzed subjects consisting of HC, MMA, and SA participants, the large proportion of severe asthmatic patients was observed in senescence low group as compared to senescence high group (*P* < 0.05, Fig. 3, G). These findings indicate that cellular senescence may be negatively associated with the severity of asthma.

### Airway CD206 ^+^ macrophages show a lower prevalence of senescence and increased cytokines

**Figure 4:**
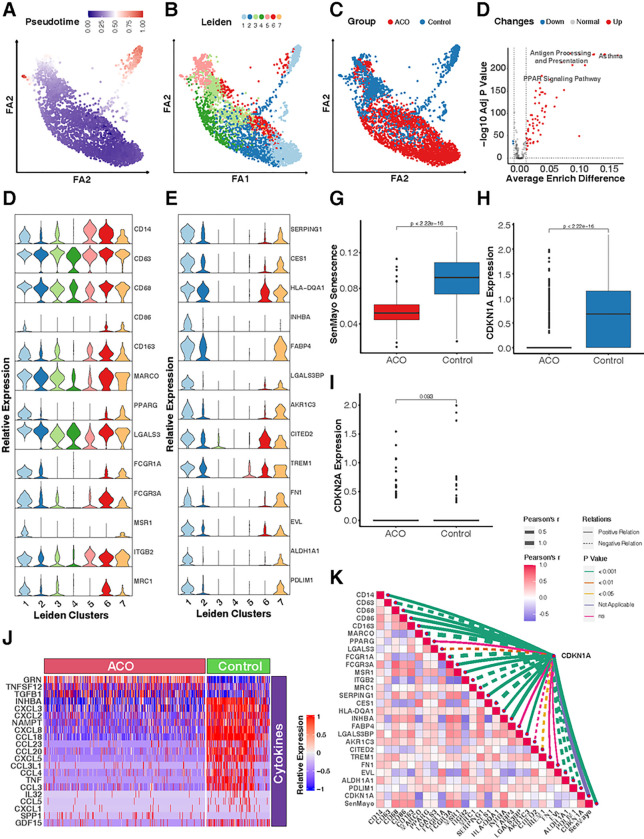
Airway CD206^+^macrophages show a lower prevalence of senescence but increased cytokines. **A,** Density scatter plots showed the distribution of relative expression of markers for cell type annotation. **B,** A total of 7 different cell clusters annotated using a combination of surface marker genes. **C,** Relative percentage of each cell type in senescence clustering low and high group. **D,** Levels of cytokines in BAL fluids in senescence clustering low and high group of the IMSA cohort. **E,** Heatmap represents cytokine expression in CD206^+^ macrophages of senescence clustering low and high group. Variables. Limma based multiple linear regressions were performed for abundance and expression comparisons between senescence high and low groups. A p < 0.05 was considered as statistical significance.

To further explore the relationship between cellular senescence and airway inflammation in asthma, we focused on the CyTOF dataset that targets lineage markers for the adaptive and innate immune systems^35^. Unsupervised cell clustering was performed in BAL fluids from HC, MMA, and SA participants using the FlowSOM algorithm^62,63^. Cell types in those clusters were determined by the surface marker staining intensities across t-SNE spaces. A total of 7 different cell clusters were annotated using a combination of surface marker genes, including B lymphocytes (CD19^+^), CD206^−^ macrophages (CD11C^+^CD206^−^), CD206^+^ macrophages (CD11C^+^CD206^−^), CD4 T lymphocytes (CD3^+^CD4^+^), CD8 T lymphocytes (CD3^+^CD8^+^), γδ T lymphocytes (TCRγδ^+^), and NK cells (CD56^+^) (Fig. 4, A, B, and Figure E3). To identify the link between these airway immune cells and cellular senescence, the percentages of those identified cell populations were compared in senescence high and low groups. Among all these populations, a significant difference was found for CD206^+^ macrophages, which showed a higher proportion in senescence low in relative to senescence high group (Fig. 4, C). Interestingly, the senescence low group showed elevated levels of cytokines IL-5, IL-13, IL-10, and IL-22 in BAL fluids of the IMSA cohort (Fig. 4, D). Particularly, CD206^+^ macrophages showed increased expression of IL-4, IL-13, and IL-22 in senescence low group as compared those in senescence high group (Fig. 4, E). Taken together, these data suggest that CD206^+^macrophages are major airway immune cells associated with cellular senescence and cellular senescence is negatively correlated with cytokines in airway CD206^+^Macrophages.

### Differentially expressed genes and pathways in Alveolar macrophages of ACO

**Figure 5:**
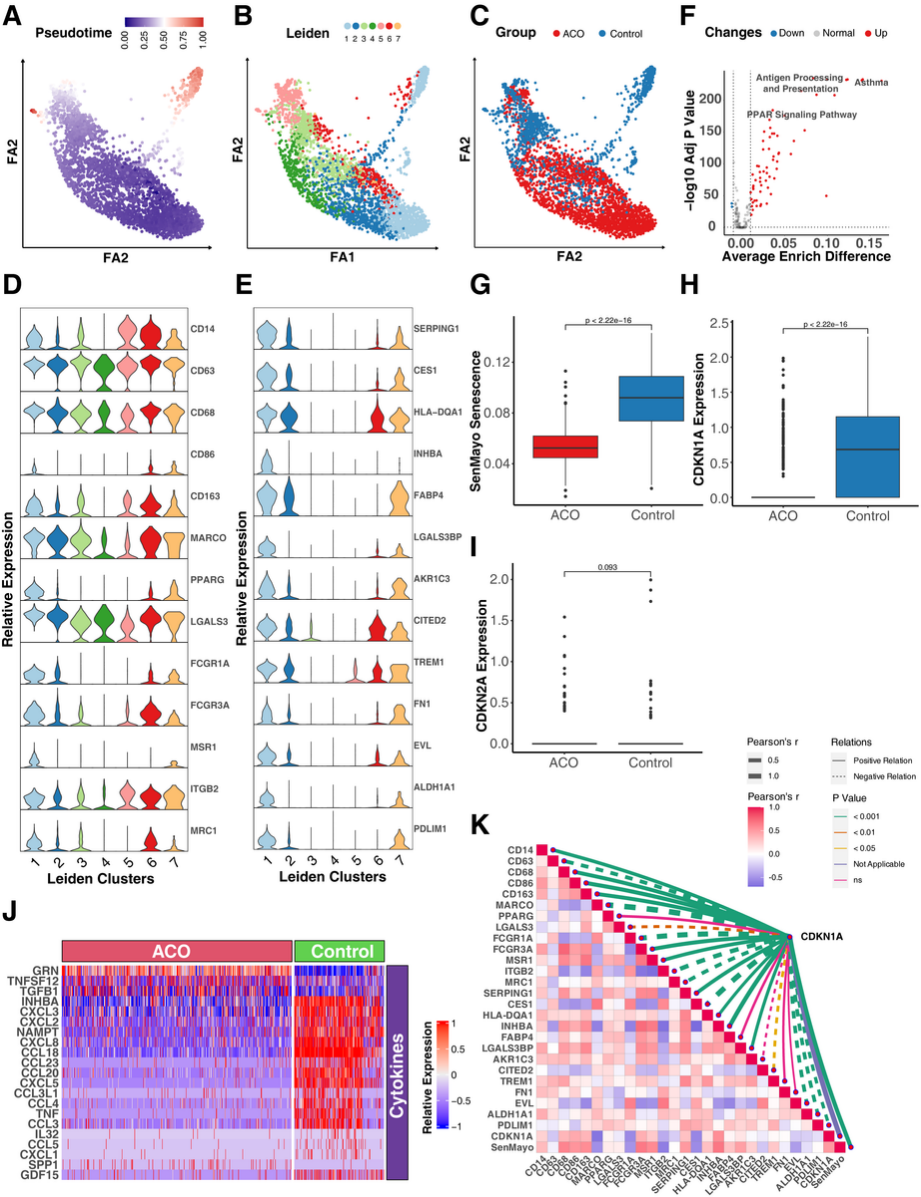
Differentially expressed genes and pathways in alveolar macrophages of ACO. **A,** A two-dimensional scatter plot of trajectory estimation of all cells from monocytes/macrophages. **B,** Distribution of cell clusters identified by Leiden matching trajectory analysis. **C,** Distribution of cells strata by groups matching trajectory analysis. **D,** Relative expression of lineage markers commonly used for monocytes/macrophages. **E,** Top-ranking high expressed marker genes across 7 cell clusters identified by FindMarkers. **F,** Difference of AUCell KEGG enrichment scores between Leiden cluster 1 and other clusters. **G,** Difference in SenMayo senescence score between ACO and control group. **H-I,** Relative expression of senescence markers CDKN1A (p21, **H**) and CDKN2A (p16, **I**) in ACO and control group. **J**, Heatmap representing the differential expression of genes linked to oxidoreductases, cytokines, and growth factors between ACO and control group. **K,** Correlations between CDKN1A expression and differentially expressed genes in Leiden cluster 1 and lineage markers for monocytes/macrophages. Wilcox rank sum tests were used for the comparison of numerical variables between 2 groups; Pearson’s correlations were used for computing coefficients between imputed expression values. A p < 0.05 was considered as statistical significance.

Given the significance of CD206^+^macrophages in cellular senescence, we re-visited the ACO dataset and determined whether the specific subtype of monocytes/macrophages drive the difference in cellular senescence between ACO and control group. To explore this, we used trajectory analysis to infer the relationships and distances between different subtypes of monocytes/macrophages. Analyses were started with cycling cells, we identified a specific cluster that has the highest distance (pseudo-time) and is in the upper right corner of a two-dimensional scatter plot (Fig. 5, A). This cluster was mostly composed of cells from Leiden cluster 1 (Fig. 5, B) and was predominantly found in the control group (Fig. 5, C), suggesting that cells in Leiden cluster 1 may contribute to the difference in biological processes between ACO and control group. To further characterize the Leiden cluster 1, we employed the FindAllMarkers to investigate the highly expressed marker genes for cluster 1 against other cell clusters. A total of 47 genes were identified specifically for the Leiden cluster 1, such as *SERPING1, CES1, HLA-DQA1, INHBA, FABP4, LGALS3BP, AKR1C3, CITED2, TERM1, FN1, EVL, ALDH1A1,* and *PDLIM1* (Fig. 5, D and see Table E2). Additionally, we checked the lineage markers that are commonly used for monocytes/macrophages (Fig. 5, E). we found that PPARγ, FCGR1A, FCGR3A, MSR1 (CD205), and MRC1 (CD206) were enriched in Leiden cluster 1. Furthermore, we used AUCell scoring to analyze the biological characteristics of Leiden cluster 1 in comparison to other clusters. Especially, the KEGG enrichment analysis (Fig. 5, F and see Table E3) indicated that Leiden cluster 1 had significant enrichments in several pathways, such as antigen processing and presentation, Peroxisome Proliferator-Activated Receptor (PPAR) signaling pathway. Next, we investigated the differences in cellular senescence between ACO and control groups specially in Leiden cluster 1. Consistent with our previous analyses among all the monocytes/macrophages, the SenMayo senescence score was remarkably lower in ACO groups compared with control group (Fig. 5, G). This was further supported by the expression of cellular senescence marker CDKN1A (Fig. 5, H), which also showed reduced expression in the ACO group. No statistical difference was observed for CDKN2A (Fig. 5, I). Additionally, to further display the characteristics of cellular senescence, we identified differentially expressed genes related to oxidation-reduction, cytokines, and growth factors between ACO and control groups in Leiden cluster 1. Of those, TGFB1 was increased but several chemokines such as CXCL2, CXCL3, CXCL8, and CCL18 were decreased in ACO group compared with control group (Fig. 5, J and see Table E4). These results suggest correlations between cellular senescence and cytokine expression in alveolar macrophages of Patients with ACO. Furthermore, strong correlations were identified for CDKN1A expression levels and those identified genes in Leiden cluster 1 and traditional lineage markers for monocytes/macrophages (Fig. 5, K and see Table E5 in this article’s Online Repository at www.jaci.org). For example, CDNK1A expression was correlated with genes with either positive regulation (e.g., *CD14, CD68, CD86, CD163, FCGR3A, MSR1, SERPING1, HLA-DQA1, INHBA, LGALS3BP, AKR1C, TREM1, FN1,* and *ALDH1A1*) or negative regulation (e.g., *MARCO, FCGR1A, ITGB2,* and *MRC1*). Collectively, the results indicate that these identified genes and biological pathways may be involved in regulating cellular senescence in alveolar macrophages of patients with ACO.

### PPARγ, a Key Regulating Factor of Cellular Senescence in Alveolar Macrophages

**Figure 6:**
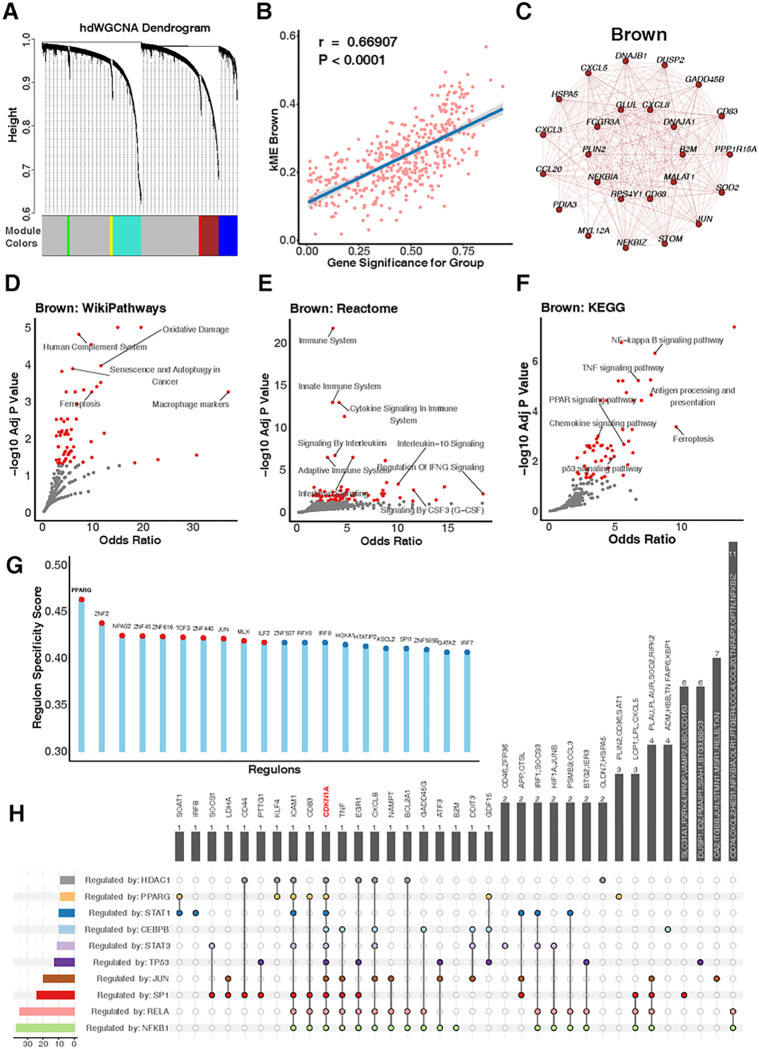
PPARγ as a key regulating factor of cellular senescence in alveolar macrophages. **A,** Six gene modules identified by the hdWGCNA analysis focused on Leiden cluster 1. **B,** Brown module was positively correlated with the difference in cellular senescence between ACO and control groups. C, Top 30 hub genes withhigh correlations in the brown module. **D-F,** Different pathways in the brown module were identified by WikiPathways (**D**), Reactome (**E**)and KCGG (**F**). **G**, Top 10 specific regulons for Leiden cluster 1 ranked by score value generated from PySCENIC. **H,** Transcription factor enrichment analysis within the brown module against the TRRUST database.

To delve deeper into the underlying regulatory mechanisms of biological changes, we first utilized the hdWGCNA analysis focused on Leiden cluster 1 to explore the expression network associated with senescence signatures. The algorithm discerned six gene modules: green, blue, red, brown, cyan, and tan (Fig. 6, A). Within these, CDKN1A (p21) was pinpointed in the brown module, and the module feature gene score was positively correlated with the difference in cellular senescence between ACO and control groups (R = 0.669, p < 0.0001, Fig. 6, B). Prominently, the top 30 hub genes with high correlation in this module encompassed those related to growth arrest and proliferation (e.g., GADD45B, PPP1R15A, and DUSP2), TP53-mediated cell senescence-associated heat shock proteins (*DNAJA1, DNAJB1,* and *HSPA5*), DNA damage and repair (e.g., MYL12A), and energy, protein, and lipid metabolism (e.g., *GLUL, PLIN2, RPS4Y1, PDIA3,* and *SOD2*). Furthermore, genes associated with SASP and its upstream regulators (e.g., *CCL20, CXCL3, CXCL5, CXCL8, JUN, NFKB1A,* and *NFKBIZ*) and those tied to antigen presentation, antibody response, and macrophage activation (e.g., *C83, CD68, FCGR3A,* and *B2M*) were also included in these hub genes (Fig. 6, C). These genes or biological processes are directly or indirectly involved in senescence processes of alveolar macrophages. Subsequent enrichment analyses of entire module genes showed that the brown module was mainly associated with macrophage markers and cellular senescence (Fig. 6, D), cytokine signaling in immune system (Fig. 6, E), and antigen processing and presentation and PPAR signaling (Fig. 6, F). These biological characteristics are closely related and mutually regulated. To pinpoint the central regulator of senescence in alveolar macrophages, we leveraged SCENIC to discern the most specific transcription factors for each Leiden cluster. Notably, for Leiden cluster 1, PPARγ emerged as the predominant regulator (Fig. 6, G). This was further supported by the transcription factor enrichment analysis with the brown module against the TRRUST database. PPARγ was identified to be one of the top 10 pivotal transcription factors modulating gene expression within this module (Fig. 6, H). Intriguingly, each of these transcription factors had the potential to regulate CDKN1A expression. Taken together, these findings indicate that the brown module had the core impact on cellular senescence, and PPARγ is one of the predominant regulators modulating the senescent signature of alveolar macrophages.

## DISCUSSION

Both asthma and COPD can co-exist in older individuals with clinically overlapping phenotypes^1–3^. ACO has recently been a focus of interest because patients with ACO often experience poor health-related quality of life, increased rates of exacerbations, and severe clinical symptoms^7,8^. While the exact pathophysiology of ACO is not fully understood, it is thought to result from various features of both asthma and COPD ^64,65^. Both asthma and COPD involve chronic airway inflammation, but they have different underlying mechanisms. In asthma, there is typically eosinophilic inflammation driven by a Th2 immune response^65^, while COPD is characterized by neutrophilic inflammation associated with Th1 and Th17-driven inflammation^10,66,67^. In both conditions, macrophages play a central role in the recruitment and activation of neutrophils and eosinophils^11–13,68^. Indeed, a very recent cross-sectional exploratory study was performed to investigate cellular changes (e.g., macrophages, neutrophils, eosinophils, mast cells, CD8^+^, and CD4^+^T lymphocytes) in the airway wall of ACO compared with asthma, COPD current smokers, and ex-smokers, normal lung function smokers, and non-smoker controls. They found that the ACO airway tissue inflammatory cellular profile differed from the contributing diseases of asthma and COPD with a predominance of macrophages^65^. The results were supported by our analyses on an existing scRNA-Seq dataset generated from human lung tissues of patients with ACO. Monocytes/macrophages were a predominant cell type among all cell type to be analyzed (monocytes/macrophages, T cells, NK cells, AT2 alveolar type II cells, endothelial cells, airway epithelial cells, B cells, fibroblasts, and mast cells), constituting more than 50% of the total cells, and the proportion of monocytes/macrophages was significantly higher in patients with ACO in relative to control group. Importantly, our further study identified several sub-types of monocytes/macrophages, including alveolar macrophages, cycling cells, interstitial macrophages, and monocytes. Of these, alveolar macrophage was the most predominant cell type with the increased expression of complement-related genes C1QA, C1QB, and several other genes RBP4, CD9, SERPING1, and CES1. Thus, our findings provide further evidence that monocytes/macrophages, particularly alveolar macrophages, are major cells that may contribute to airway inflammation in patients with ACO.

Next, we explored how monocytes/macrophages drive airway inflammation with the emphasis on cellular senescence that has been implicated in the pathophysiology of various diseases, including asthma^29^. Cellular senescence is an extremely complex and dynamic biological process induced by several factors, including aging, oxidative stress, DNA damage, mitochondrial dysfunction, epigenetic modifications, and telomere shortening^29,69,70^. Cellular senescence has been associated with both asthma^29^ and COPD^30–32^. Several stimuli causing cellular senescence have been associated with asthma, such as telomere shortening, oxidative stress, inflammation, and autophagy/mitophagy. Therefore, senescence may exert a significant influence on the function and activation of ACO-associated target cells and subsequently development and management of ACO. Here we investigated the relationship between senescence and ACO by analyzing gene signatures associated with senescence in all the subtypes of monocytes/macrophages. We demonstrated for the first time a lower prevalence of cellular senescence in alveolar macrophages of patients with ACO. Furthermore, CDKN1A (p21) but not CDKN2A (p16) showed lower expression in alveolar macrophages of patients with ACO, suggesting that CDKN1A (p21) pathway may play a major role in mediating senescence of alveolar macrophages in ACO.

To strengthen our findings on the role of cellular senescence in ACO, we analyzed the relationship of cellular senescence features with the severity of asthma in a total of 39 individuals from IMSA^35^. Specifically, senescence in those individuals were clustered into senescence low and high group and then the distribution of healthy controls, mild/moderate asthma, and severe asthma was analyzed in these two groups. Intriguingly, the large proportion of severe asthmatic patients was observed in senescence clustering low group compared with senescence clustering high group. Furthermore, we focused on the CyTOF dataset from BAL fluids that targets lineage markers and identified a total of 7 different cell clusters, including B lymphocytes, CD206^−^ macrophages, CD206^+^ macrophages, CD4^+^ T, CD8^+^ T, γδ T, and NK cells. Of these, CD206^+^ macrophages were distributed in senescence clustering low group and showed increased expression of Th2 cytokines IL-4, IL-13 and Th17 cytokine IL-22, suggesting that, CD206^+^ macrophages may drive both Th2 and Th17 populations commonly seen in patients with ACO^64,65^. CD206 expression is a characteristic feature of some tissue-resident macrophages, including alveolar macrophages^11,71^, and CD206^+^ macrophages represent a large proportion of alveolar macrophages. Collectively, these independent data provide evidence that patients with severe asthma have a lower prevalence of cellular senescence, and CD206^+^macrophages may contribute to the severity of asthma, possibly ACO, by releasing inflammatory cytokines.

We also explored the underly mechanisms as for how cellular senescence is regulated in macrophages. We investigated the differentially expressed genes and pathways in alveolar macrophages in Leiden cluster 1, a population mostly likely contributing to the difference in cellular senescence between ACO and control group. Several pathways were also identified, such as antigen processing and presentation, PPARγ signaling pathway. Additionally, several markers commonly used for monocytes/macrophages also showed different expression, including PPARγ, FCGR1A, FCGR3A, CD205, and CD206. As expected, most of these differentially expressed genes were either positively or negatively associated with senescence as defined by the expression of CDNK1A and enrichment scores of SenMayo senescence (data not shown). To further delve deeper into the underlying regulatory mechanisms of biological changes, we utilized the hdWGCNA analysis focused on alveolar macrophages and identified several expression networks associated with senescence signatures, including growth arrest and proliferation, TP53-mediated cell senescence-associated heat shock proteins, DNA damage and repair, and energy, protein, and lipid metabolism. These genes or biological processes are directly or indirectly involved in senescence processes of alveolar macrophages.

Importantly, we identified PPARγ as one of the top 10 pivotal transcription factors modulating senescent signatures of alveolar macrophages. Interestingly, PPARγ as a transcription factor has been shown to play a crucial role in various physiological processes, including adipogenesis^72,73^, glucose homeostasis^74^, inflammation^75,76^, and metabolism and function of macrophages^77^. Studies have implicated that PPARγ regulates senescence in various cell types, including fibroblasts and endothelial cells^78,79^, and macrophages^77^. PPARγ activation can promote senescence by upregulating the expression of specific senescence-associated genes and inducing cell cycle arrest^79^. PPARγ activation can also have anti-senescent effects by reducing senescence and improve metabolic function^79–82^

Taken together, we present groundbreaking findings, demonstrating for the first time that monocytes/macrophages, particularly alveolar macrophages, are the predominant cell types in patients with ACO. Our research reveals a lower prevalence of senescence within alveolar macrophages in both ACO patients and those with severe asthma. Mechanistically, our in-depth exploration of differentially expressed genes within alveolar macrophages has identified PPARγ as a crucial regulatory factor driving senescence in ACO. Notably, there are several limitations, including small sample size, lack of mouse model to mimic patients with ACO, and complicated and context-dependent role of PPARγ in cellular senescence. However, our observations in this study provide a solid foundation for future investigations aimed at a comprehensive understanding of the mechanisms through which PPARγ regulates senescence. Furthermore, it opens doors for exploring PPARγ as a potential therapeutic target for interventions aimed at modulating senescence-associated processes in ACO -a condition marked by mixed features of both asthma and COPD, known for its increased severity.

## Data Availability

All bioinformatical data can be downloaded from figshare database (scRNA-Seq from fatal ACO and two health control: https://doi.org/10.6084/m9.figshare. 14782377.v1.), GEO (Bulk RNA-Seq data from IMSA BALF: GSE136587) database and flow repository database (CyTOF data from IMSA BALF: FR-FCM-Z395).

## Abbreviations

IL: Interleukins
AM: Alveolar Macrophages
IM: Interstitial Macrophages
SASP: Senescence Associated Secretory Phenotype
COPD: Chronic Obstructive Pulmonary Diseases
ACO: Asthma-COPD Overlap
CyTOF: Cytometry by Time of Flight
BALF: Bronchoalveolar Lavage Fluid
IMSA: Immune Mechanisms Severe Asthma
UMAP: Uniform Manifold Approximation and Projection
DEGs: Differential Expressed Genes
H&E: Hematoxylin and Eosin
SCENIC: Single-Cell rEgulatory Network Inference and Clustering
hdWGCNA: High-Dimensional Weighted Gene Co-epression Network Analysis
PAGA: Partition-Based Graph Abstraction
GEO: Gene Expression Omnibus
GSEA: Geneset Enrichment Analysis
TRRUST: Transcriptional Regulatory Relationships Unraveled by Sentence-based Text ` mining
ssGSEA: Single Sample GSEA
CRE: Cockroach Extract
KEGG: Kyoto Encyclopedia of Genes and Genomes
GO: Gene Ontology
PPARγ: Peroxisome Proliferator Activated Receptor Gamma
